# Impact of osteotomy angle on bone failure risk in a modified pull-through approach: a finite element analysis

**DOI:** 10.1186/s12903-025-06732-6

**Published:** 2025-09-08

**Authors:** Vincenzo Orassi, Philipp Ruf, Elena Hofmann, Steffen Koerdt, Kilian Kreutzer, Susanne Nahles, Max Heiland, Carsten Rendenbach, Sara Checa, Norbert Neckel

**Affiliations:** 1 Berlin Institute of Health at Charité – Universitätsmedizin Berlin, Julius Wolff Institute, Berlin, Germany; 2 Department of Oral and Maxillofacial Surgery, Charité – Universitätsmedizin Berlin, corporate member of Freie Universität Berlin, Humboldt-Universität zu Berlin, Berlin, Germany; 3 BIH Biomedical Innovation Academy, Berlin Institute of Health at Charité- Universitätsmedizin Berlin, Berlin, Germany; 4 Berlin-Brandenburg School for Regenerative Therapies, Berlin, Germany; 5 Institute of Biomechanics, TUHH Hamburg University of Technology, Hamburg, Germany

**Keywords:** In Silico model, Osteotomy angle, Mandibular lingual release, Oral cancer, Oropharyngeal cancer, Fracture risk, Pull-through technique

## Abstract

**Background:**

A modified pull-through approach represents a promising treatment strategy to access tumors in the posterior oral cavity. The design of the wedge osteotomy plays a key role in preserving postoperative mechanical stability while enabling surgical access. However, the optimal osteotomy design to reduce fracture risk remains unclear. Therefore, this study aimed to test osteotomy wedge designs that have the potential to lower the bone fracture risk.

**Methods:**

Four wedge osteotomy configurations were compared using finite element analysis based on a realistic mandible model. Each design differed in the angles and curvature of the osteotomy planes. Unilateral molar clenching was simulated, and mechanical strains were quantified and compared to the yield strain of cortical bone in the canine region to evaluate the risk of bone failure.

**Results:**

The finite element analysis showed that a wedge osteotomy with less acute angles in the canine region has a lower fracture risk when compared to osteotomies with sharp angles. Peak bone strain values could be reduced by half by changing the osteotomy angle at the canine region.

**Conclusions:**

A larger angle between the osteotomy cutting planes offers mechanical advantages by reducing strain concentrations in critical regions. These findings provide valuable guidance for refining the current surgical technique and support the integration of biomechanical analyses into osteotomy planning to optimize surgical outcomes.

**Supplementary Information:**

The online version contains supplementary material available at 10.1186/s12903-025-06732-6.

## Introduction

Cancer resection in the posterior oral cavity or oropharynx inherently presents difficulties regarding surgical accessibility. This can affect the tumor resection with appropriate and clear resection margins and the subsequent reconstruction of the defect [1].

Therefore, several surgical approaches are employed to address this issue [2]. Transoral robotic surgery is a recent development that can mitigate some of these challenges, but it has various limitations concerning general accessibility and applicability, restricting its use to a limited number of centers worldwide [1–3]. Potential conventional alternatives are the mandibular split and mandibular lingual release [4]. Both techniques have several inherent side effects. In the conventional mandibular lingual release, these are primarily due to a potentially insufficient reconstruction of the anatomically correct muscle attachment of the hyoid bone and tongue, which can lead to impaired swallowing and speech [1, 4, 5]. The mandibular split approach additionally carries the risk of occlusal inaccuracies and malunion [1, 4, 5].

To address this, we introduced a modification of the mandibular lingual release or pull-through technique and an alternative to the mandible-split technique [1], utilizing virtually designed cutting guides. A key element of this surgical approach is a pyramid- or wedge-shaped chin bone flap, pedicled to the genioglossus and geniohyoid muscles, which allows for self-refixation due to a tapering mental cutout and the pulling force applied by the intact musculature after tumor resection. This shape is intended to facilitate dynamic compression of the osteotomy lines by the muscle tone at baseline, thereby eliminating the need for the implantation of osteosynthesis material. This principle supports early functional loading and is considered to enhance early functional rehabilitation. Even though we deem this procedure to be safe and clinically feasible, the absence of internal fixation presents an inherent risk of compromising the mechanical stability of the mandible in a critical area, as a lower vertical depth of the corpus correlates with higher tensile and shear stresses [1, 6].

A significant drawback of the proposed osteotomy is its shape, since the potential proximity to the canine roots and sharp edges in this area might increase the risk of pathological fractures. In particular, due to the long roots of these teeth, this location represents a predetermined breaking point, as teeth are suspended in the alveolus by Sharpey’s fibers, which do not enhance stability [7, 8]. Such a postoperative fracture can delay functional rehabilitation and may postpone necessary adjuvant treatments, thereby undermining the advantages of this surgical approach.

In this study, we assess bone failure risk associated with different variations in osteotomy shape for the modified pull-through approach by employing computational modeling techniques. In particular, finite element analysis (FEA) offers a powerful computational approach to evaluate key parameters otherwise challenging to quantify in vivo or in experimental settings [6, 9, 10]. By comparing stress and strain distributions within the mandibular bone under a physiological clenching task, FEA allows a direct investigation of the biomechanical environment induced by each osteotomy design in the same subject and under the same loading conditions.

## Methods

### Finite element models

A computed tomography (CT) scan of a dentate mandible from a 58-year-old male subject was retrieved, with a voxel size of 512 × 512, and an in-plane pixel spacing of 0.625 mm x 0.625 mm (Aquilion PRIME, Canon Medical Systems, Otawara, Tochigi, Japan). The mandible was segmented using Mimics 25.0 (Materialise NV, Leuven, Belgium), differentiating between cortical and trabecular bone and teeth. These were then imported as solids into the finite element software Abaqus (Dassault Systèmes Simulia Corp., United States).

### Material properties

**Fig. 1:**
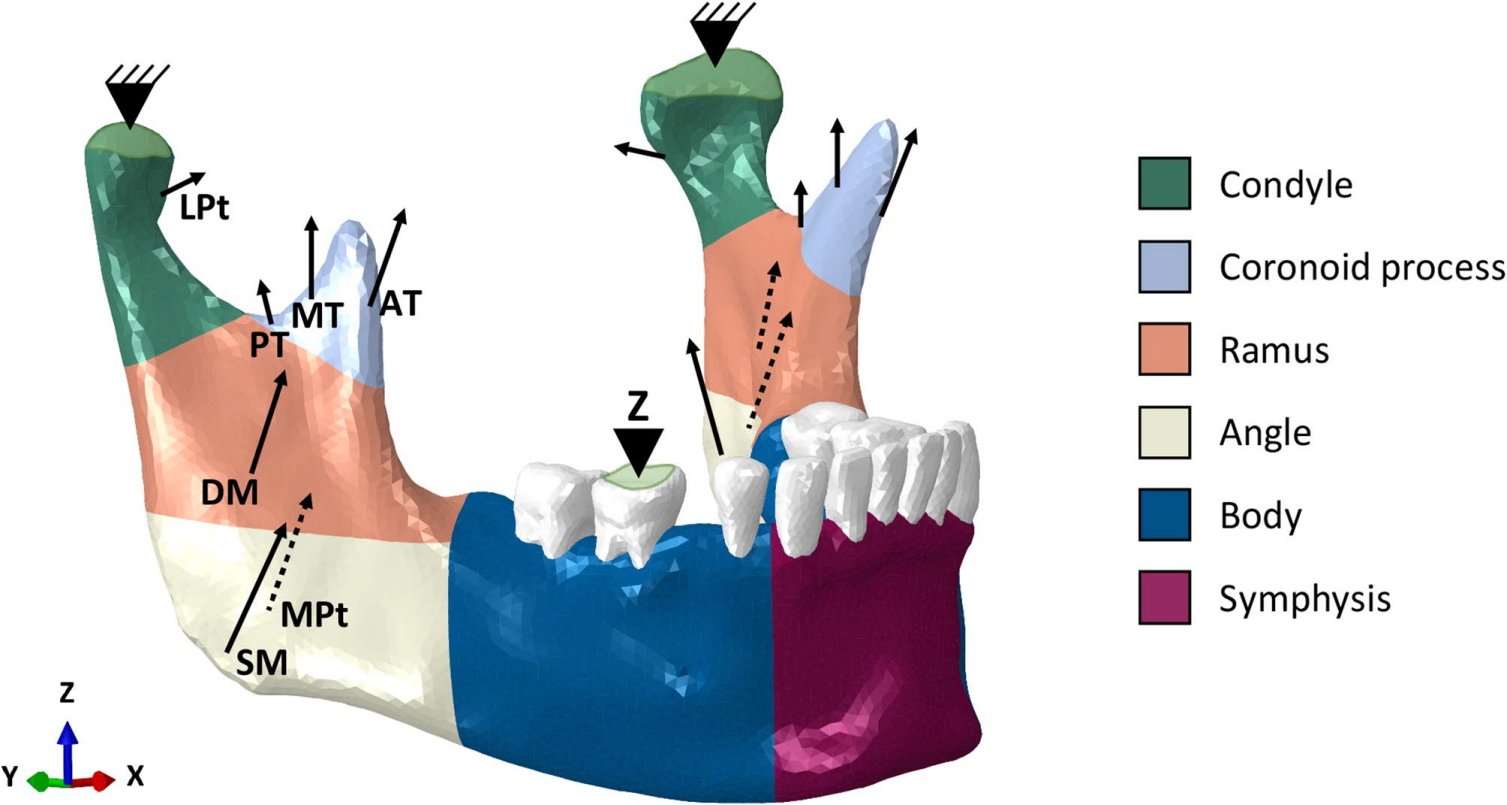
Orthotropic sections, loading, and boundary conditions in the finite element model. Orthotropic cortical bone regions are depicted with different colors. Muscle forces responsible for closing the mandible are represented as vectors; a right unilateral static clenching was simulated by restraining both the condyles movement in the 6 degrees of freedom, as locked in the glenoid fossa, and the vertical displacement (Z direction) of the right first molar at the occlusion

Orthotropic material properties were assigned to the cortical bone [11], reflecting the spatial variation in bone structure across six distinct regions of the mandible – symphysis, body, angle, ramus, coronoid, and condyle (Fig. 1). The elastic and shear moduli and Poisson’s ratios for each region were defined as described by previous studies [9, 12]. Trabecular bone [13] and teeth [12] were modeled as isotropic materials. All materials are reported in Table 1.

**Table 1:** Material properties of cortical bone, trabecular bone, and teeth. Orthotropic properties were assigned to six distinct regions of the cortical bone. Isotropic properties were assigned to trabecular bone and teeth

Material properties	Cortical bone [9, 12]						Trabecular bone [13]	Teeth [12]
	Symphysis	Body	Angle	Ramus	Condyle	Coronoid process		
Ex	20,492	21,728	23,793	24,607	23,500	28,000	300	17,600
Ey	16,350	17,828	19,014	18,357	17,850	17,500	300	17,600
Ez	12,092	12,700	12,757	12,971	12,650	14,000	300	17,600
νxy	0.34	0.34	0.3	0.28	0.24	0.23	0.3	0.34
νyz	0.22	0.2	0.22	0.23	0.25	0.28	0.3	0.34
νxz	0.43	0.45	0.41	0.38	0.32	0.28	0.3	0.34
Gxy	6908	7450	7579	7407	7150	7150	-	6567
Gyz	4825	5083	4986	5014	5150	5300	-	6567
Gxz	5317	5533	5493	5386	5500	5750	-	6567

*E* Elastic modulus (MPa), *ν* Poisson’s ratio (-), *G* Shear modulus (MPa)

### Loading and boundary conditions

Loading conditions replicated the forces acting on the mandible during unilateral right-side clenching post-operatively. The considered muscle groups were the superficial (SM) and deep (DM) masseter, anterior (AT), medial (MT), and posterior (PT) temporalis, and medial (MPt) and lateral (LPt) pterygoid muscles (Fig. 1). Maximum force magnitudes were applied as the worst (most challenging) biomechanical scenario [6]. A bite force was simulated by constraining the vertical displacement at the occlusal surface of the first molar and by constraining both condyles in all six degrees of freedom to mimic the fixed position of the temporomandibular joints during clenching (Fig. 1). Muscle attachments, direction cosines, and activation patterns for each muscle as well as boundary conditions were assigned based on Korioth et al. [6] (Table 2).

**Table 2:** Magnitudes, directions, and activation patterns of the masticatory muscle forces during right unilateral clenching [6]

Muscle groups	Maximum muscle force (*N*)	Direction cosine (-)				Fiber activation (-)	
		X		Y	Z	Unilateral clenching	
		Right	Left			Right	Left
Superficial Masseter	190.4	−0.207	0.207	−0.419	0.884	0.72	0.60
Deep Masseter	81.6	−0.546	0.546	0.358	0.758	0.72	0.60
Anterior Temporalis	158.0	−0.149	0.149	−0.044	0.988	0.73	0.58
Medial Temporalis	95.6	−0.222	0.222	0.500	0.837	0.66	0.67
Posterior Temporalis	75.6	−0.208	0.208	0.855	0.474	0.59	0.39
Medial Pterygoid	174.8	0.486	−0.486	−0.373	0.791	0.84	0.60
Lateral Pterygoid	66.9	0.630	−0.630	−0.757	−0.174	0.30	0.65

### Design of the wedge fragments

To simulate the modified pull-through approach proposed earlier [1] and test new wedge designs, four different mental osteotomies were virtually designed (KLS Martin GmbH, Tuttlingen, Germany). Specifically, the wedge designs can be distinguished by the number and type of cutting planes used:

- Wedge 1 (W1), three straight cutting planes [1].
- Wedge 2 (W2), four straight cutting planes.
- Wedge 3 (W3), two straight cutting planes and one wide-arched cutting plane.
- Wedge 4 (W4), two straight cutting planes and one acute-arched cutting plane.

**Fig. 2:**
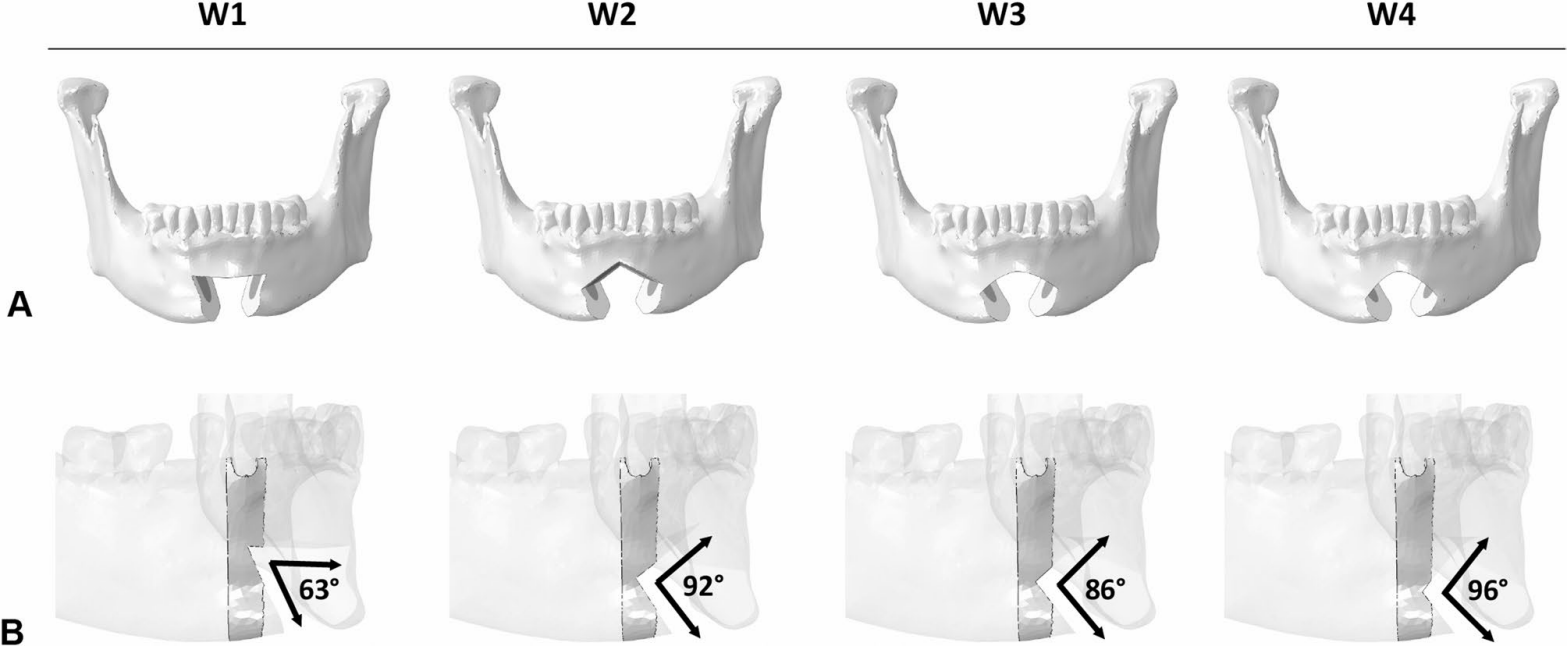
Overview of the wedge designs and regions of interest. (**A**) Wedge osteotomy designs. (**B**) Regions of interest (ROIs) at the right canines (solid color) and relative angles between the osteotomy cutting planes

W1, W2, W3, and W4 designs are reported in Fig. 2A.

In all cases, only the osteotomized mandible was included in the analysis, while the wedge segment was omitted to reflect the immediate post-operative condition, in which it is held in place by surrounding muscles but does not provide mechanical support to the mandible.

### Analysis

A maximum principal strain criterion [14] was employed to estimate the failure risk of the cortical bone across the four wedge designs during the early post-operative phase. In particular, the analysis focused on the maximum principal strain (ε_max_) to identify regions with peak tensile loading, and the maximum shear strain (γ_max_) (Eq. 1) to account for tissue distortion.

Every right canine region, hereafter referred to as the region of interest (ROI), was characterized by a different osteotomy angle (Fig. 2B). A fracture risk assessment was performed by normalizing both ε_max_ and γ_max_ with respect to their yield values (ε_yield_ = 0.45% and γ_yield_ = 0.57% [15]) (Eqs. 2 and 3) within the ROI, in all four scenarios [16].

1 $$\gamma_{max}=\frac12max(\vert\varepsilon_{max}-\varepsilon_{mid}\vert,\vert\varepsilon_{mid}-\varepsilon_{min}\vert,\vert\varepsilon_{min}-\varepsilon_{max}\vert)$$

2 $$\:\stackrel{-}{\epsilon\:}=\:\frac{{\epsilon\:}_{max}}{{\epsilon\:}_{yield}}$$

3 $$\:\stackrel{-}{\gamma\:}=\:\frac{{\gamma\:}_{max}}{{\gamma\:}_{yield}}\:$$

$\:{\epsilon\:}_{max}{,\epsilon\:}_{mid},{\epsilon\:}_{min}$: Normal principal strain components;

$\overline\varepsilon$: Normalised maximum principal strain;

$\overline\gamma$: Normalised maximum shear strain.

### Mesh convergence study

The models were meshed using quadratic tetrahedral elements. A mesh convergence analysis was performed for every case at the symphysis region to ensure the numerical accuracy of the finite element simulations. Mesh sizes ranging from coarse to fine were tested, and the results were evaluated based on the median values of the absolute maximum principal stress and strains within the whole cortical symphysis. The mesh sizes with a relative error of less than 2% compared to the finest mesh were selected for analysis. Specifically, in the final meshes, in the symphysis region, the edge lengths were generally inferior to 0.5 mm, and the number of elements was 313,552 for W1, 480,849 for W2, 520,714 for W3, and 515,031 for W4. The detailed convergence study is reported in the Supplementary Data.

## Results

### Mechanical strains within the symphysis region under different osteotomy configurations

**Fig. 3:**
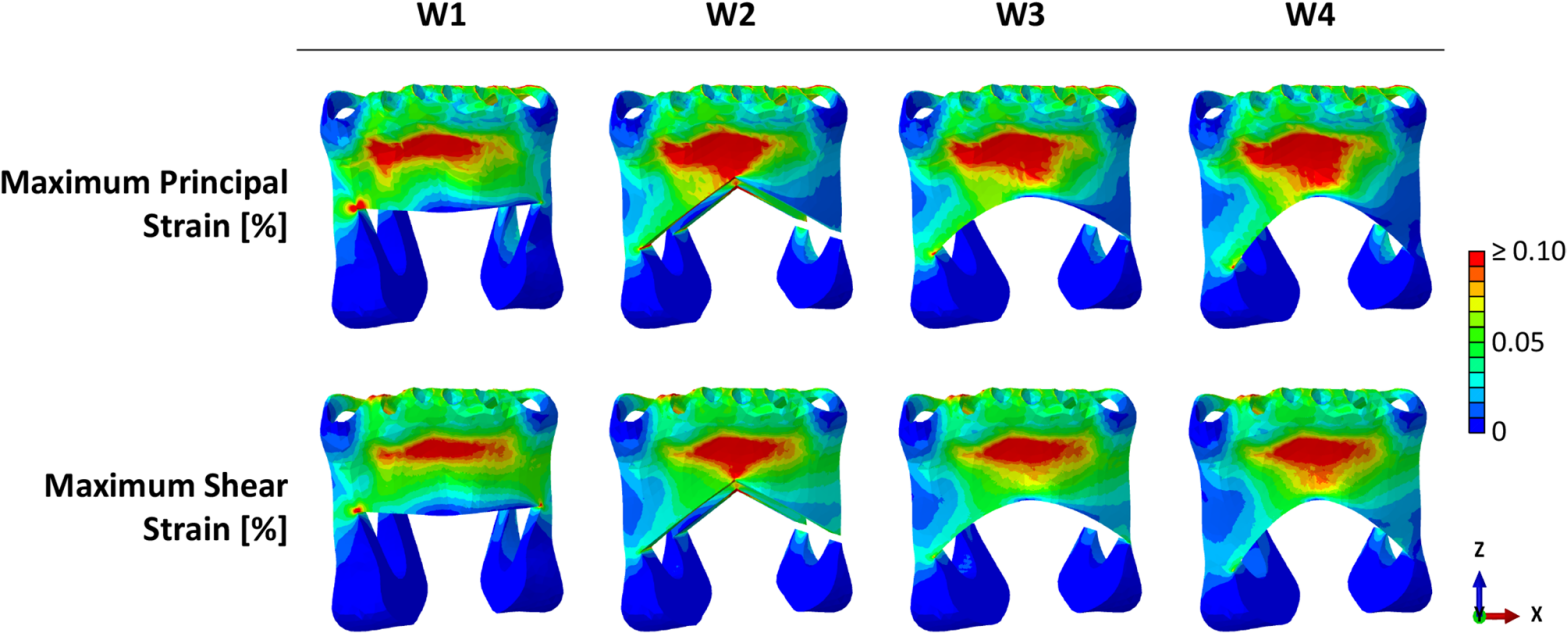
Maximum principal (ε_max_) and maximum shear (γ_max_) strain distribution within the symphysis

Maximum principal strains were calculated within the symphysis cortical region in the four osteotomy scenarios (Fig. 3). Qualitatively, the central region of the symphysis was characterized by relatively high tensile strains, comparable among all wedge designs, with W2 showing the highest strain concentration at the osteotomy cusp. At the right junction of the osteotomy planes, W1 showed the highest strains compared with the other three designs.

Similarly, the maximum shear strain distribution mirrored the previous patterns, with W1 showing peak strains at the osteotomy junction in the right canine area.

### Local strain distribution in the right canine region as an indicator of bone failure

**Fig. 4:**
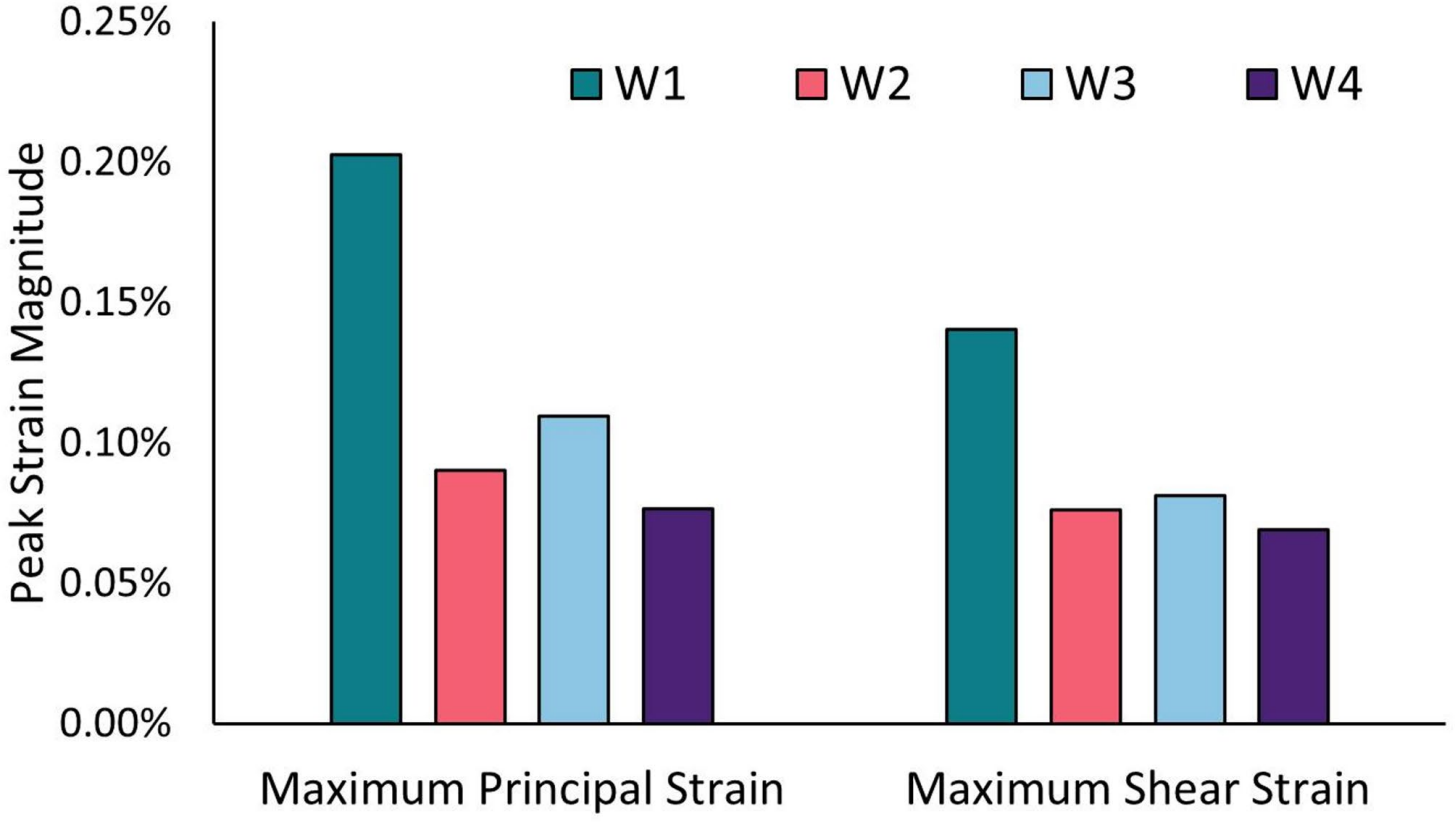
Quantification of peak maximum principal (ε_peak_) and maximum shear (γ_peak_) strain components within the ROIs

For every wedge design, the peak strains within the ROIs were quantified as the median of the 0.1% highest values to minimize the influence of local mesh artifacts (Fig. 4).

W1 showed approximately double the strain values compared to the other scenarios. Specifically, W1 exhibited the highest maximum principal strain (0.203%), while W4 showed the lowest maximum principal strain (0.077%). Intermediate values were found for W2 (0.090%) and W3 (0.109%). Similarly, W1 exhibited the highest maximum shear strain (0.141%), while W4 showed the lowest (0.069%). Intermediate values were found for W2 (0.076%) and W3 (0.081%).

**Fig. 5:**
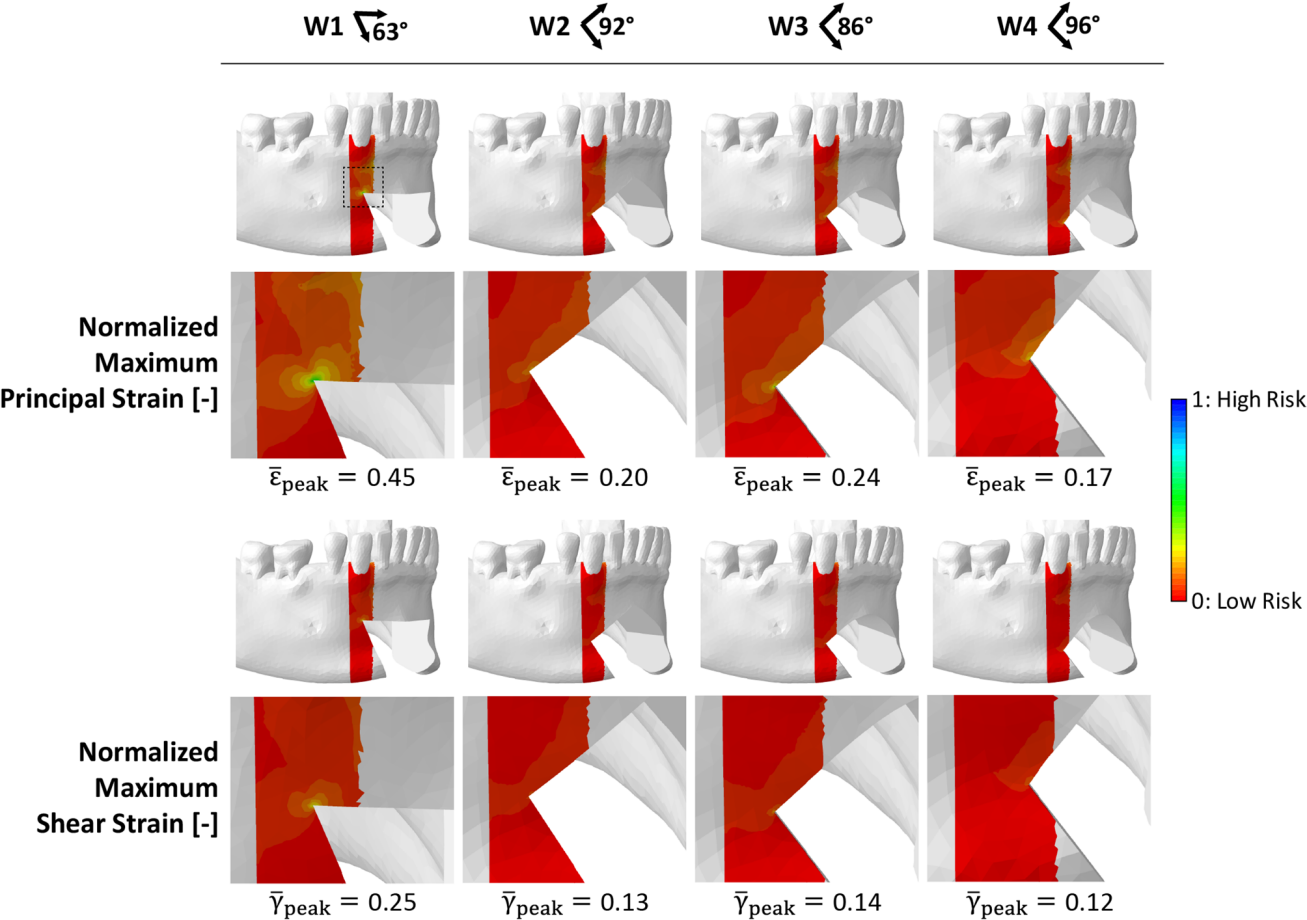
Normalized maximum principal and maximum shear strain within the ROIs. Maximum principal and maximum shear strain components normalized to the cortical bone tensile and shear yield strain, respectively, within the ROIs in the four osteotomy cases. Low and high fracture risk are associated with values close to 0 and 1, respectively

Strain values closer to the yield point resulted in normalized values near 1, suggesting an increased fracture risk. All strain values were below both tensile and shear yield strain values of cortical bone (Fig. 5). In the ROIs, normalized peak values were mainly concentrated at the osteotomy junction. More acute angles between the osteotomy planes were associated with higher normalized strain values, thus higher fracture risk. The highest normalized peak strains were found in W1 ($\:{\stackrel{-}{\epsilon\:}}_{peak}=$0.45, $\:{\stackrel{-}{\gamma\:}}_{peak}=$0.25, angle: 63°), while progressively lower strains were found in W3 ($\:{\stackrel{-}{\epsilon\:}}_{peak}=$0.20, $\:{\stackrel{-}{\gamma\:}}_{peak}=$0.13, angle: 86°), W2 ($\:{\stackrel{-}{\epsilon\:}}_{peak}=$0.24, $\:{\stackrel{-}{\gamma\:}}_{peak}=$0.14, angle: 92°), and W4 ($\:{\stackrel{-}{\epsilon\:}}_{peak}=$0.17, $\:{\stackrel{-}{\gamma\:}}_{peak}=$0.12, angle: 96°).

## Discussion

Traditional surgical methods for accessing posterior tongue and oropharyngeal tumors present several drawbacks and postoperative complications, potentially leading to non-unions and compromised masticatory functions, and resulting in aesthetic concerns [3–5]. A modified pull-through approach was proposed to overcome these limitations [1]. However, the self-retentive wedge osteotomy creates an iatrogenic weak point in the mandibular symphysis that increases the risk of pathologic fractures [17]. Biomechanically, the symphysis region has to absorb or compensate for significant bending, torsion, and shear stresses [18]. Yet, a wedge osteotomy potentially leads to an increased susceptibility of the symphysis to fatigue crack propagation [19]. Therefore, the osteotomy design plays a key role in promoting mechanical stability and reducing the risk of pathologic fractures. In this study, we evaluated variations of the previously proposed wedge osteotomy design [1] to identify features associated with reduced fracture risk.

Four wedge osteotomy designs (W1–4) for a modified pull-through approach in the mandibular symphysis were compared by assessing mechanical strain distributions during right-sided unilateral clenching in a maximum bite force condition. The cortical bone at the right canine was designated as the region of interest for quantifying the mechanical environment, as the intersection of the osteotomy planes here, the presence of the long canine roots, and the resulting thinning of the overlying cortical layer may create weak points prone to crack initiation. A maximum principal strain failure criterion [14] was chosen to identify areas experiencing peak tensile and shear loading, which are the primary failure mechanisms for cortical bone tissues [19]. Maximum principal strain and maximum shear strain in the ROIs were quantified and compared to their respective tensile and shear yield strain values [15]. The results suggest that osteotomy designs with less acute angles (e.g., W4) are biomechanically more advantageous since they lower tensile and shear strain concentrations at the right canine region, due to a smoother transition between the osteotomy planes.

Reported values of yield strain in tension and shear are 0.45% ± 0.05% and 0.57% ± 0.03% [15]. In this study, in the ROIs, W1 consistently exhibited the highest strain values ε_peak_ = 0.20% and γ_peak_ = 0.14%. On the other hand, W4 consistently showed the lowest values ε_peak_ = 0.08% and γ_peak_ = 0.07%, respectively, 62% and 51% lower than W1. Overall, the strain values in this study were always below the reported yield thresholds, suggesting that none of the osteotomy configurations are at immediate risk of failure under the simulated physiological loading conditions. However, the stress and strain concentrations in W1 at the intersection of the straight bone cuts represent a critical point for damage accumulation and fatigue failure. W4 showed the most favorable mechanical response thanks to the larger angle (96°) between osteotomy planes, compared to the 63° of W1. The strain concentration reduction was also noticeable in W2 and W3, which had angles of 92° and 86°, respectively. The osteotomy angle plays, therefore, a fundamental role in the distribution of strain within the bone. Our results well align with Bujtar et al. [20] who showed that a beveled, rather than a right-angled, osteotomy cut helps reduce the stress at the osteotomy cut. This principle has also been previously applied in structural engineering. For example, Mattheck showed how a smoother transition in a fillet improved the fatigue Life of a part by up to 40 times [21].

The wedge osteotomy designs proposed in this study represent a progressive refinement of the original W1 design [1]. The rationale underlying these modifications was to introduce smoother curvatures to help lower the cutting point in the canine region, which also provides a greater distance between the osteotomy and the dental roots. This aimed to preserve a greater symphyseal cross-sectional area, while still allowing surgical access to the posterior oral cavity. Although the arch-shaped osteotomies W3 and W4 helped reduce the strain in the middle of the symphysis compared to W2, the main reason for lower strains in the canine region was the less acute osteotomy angle in W2 and W4. Thus, this seems to be the most critical parameter when designing the wedge osteotomy. The performance of the wedge osteotomy and the associated weakening of the mandible at the symphysis should be carefully evaluated, especially if stabilizing osteosynthesis material is omitted. It must be noted that such a surgical approach still coincides with a decrease in stability and a risk of fracture. This is why the edentulous highly atrophic mandible is generally considered a contraindication for this approach [1]. However, preoperative biomechanical studies may potentially permit the application of the modified pull-through approach in individual cases presenting with an edentulous jaw with sufficient bone height.

Since the form of the osteotomy needs to be individualized, no single shape can be considered a universally optimal solution. With this in mind, a compromise between the biomechanically optimized shape and surgical feasibility must be identified based on virtual planning and biomechanical assessment for each individual situation. Overall, a patient-specific modification of the shape of the original osteotomy of the modified pull-through technique (W1) [1] is recommended. Reducing the segment size and thus the functional gap generally helps reduce the risk of fracture.

While this study provides valuable insights into the biomechanical behavior of different osteotomy designs, several limitations must be acknowledged.

A key limitation is that only four wedge designs were tested in a single patient-specific model, representing a preliminary analysis based on surgically feasible configurations. While this approach allowed for a controlled comparison and identification of design principles, such as avoiding acute angles and preferring rounded transitions, generalization to broader anatomical and surgical conditions (e.g., edentulous mandibles, mandibular atrophy, variable bone densities) remains limited. Future work should incorporate parametric variation or computational optimization techniques to identify potentially better-performing configurations to develop patient-specific osteotomy planning tools.

The chin wedge was omitted from the model to simulate a worst-case post-operative scenario, in which no osseous union is present and the wedge provides no mechanical support. As the healing progresses, the mineralization of the interface tissue between the mandible and the wedge increases the load-bearing contribution of the wedge, thereby lowering strain concentrations at the osteotomy junctions. By focusing on an immediate post-operative condition, the analysis allowed testing the most critical mechanical scenario in all osteotomy cases. The material properties used in the finite element model were assumed homogeneous and elastic, whereas bone exhibits heterogeneous properties. This is, however, a reasonable simplification considering that the study focused on the comparison of the performance of different osteotomy designs. Additionally, only static biting was included in the analysis, while in vivo conditions involve dynamic and cyclic loading. However, since all models use the same material properties and loading and boundary conditions, the relative differences in stress and strain remain valid. Furthermore, only unilateral clenching was tested, but evidence shows that this represents the most mechanically solicited scenario [9, 22] and clinically the most Likely to occur post-surgery. All designs were moreover tested in a maximum bite force condition as the worst-case scenario, with a bite force at the occlusion equal to 330 N. Previous studies on mandibular fractures and reconstruction have reported maximum bite forces of approximately 100 N during early recovery [23, 24]. However, unlike in those cases, in the modified pull-through approach, the mandibular continuity is preserved, which may allow for greater functional loading. Moreover, involuntary high bite forces (i.e., bruxism) remain clinically plausible and pose additional risk in the immediate post-operative phase. Therefore, in this study, the upper-bound load was used to ensure a conservative assessment of bone failure risk, allowing a coherent comparison across the different cases. As other FEA analyses have shown, a closed mouth position can decrease the fracture risk in the symphysis area and provide the maximum passive traction to the muscle, forcing the bony segment into its socket [22]. Therefore, a postoperative intermaxillary fixation with elastics might help to avoid unfavorable forces and outcomes. Future studies should also consider incorporating patient-specific bite force measurements to improve the clinical validation of finite element predictions.

## Conclusions

In conclusion, a biomechanical evaluation of four variations of a mandibular wedge osteotomy was performed by comparing mechanical strain distributions within the symphysis, particularly in the canine region. The results showed that designs with smoother, less acute osteotomy angles considerably reduced the peak tensile and shear strains. Preoperative biomechanical planning of osteotomy designs ensures the right compromise between the postoperative safety and feasibility of the surgical procedure.

## Supplementary Information

Supplementary Material

## Data Availability

The datasets used and/or analysed during the current study are available from the corresponding author on reasonable request.

## Abbreviations

FEA: finite element analysis
SM: superficial masseter muscle
DM: deep masseter muscle
AT: anterior temporalis muscle
MT: medial temporalis muscle
PT: posterior temporalis muscle
MPt: medial pterygoid muscle
LPt: lateral pterygoid muscle
W1: Wedge 1
W2: Wedge 2
W3: Wedge 3
W4: Wedge 4
ROI: region of interest

## References

[1] Neckel N, Neckel PH, Hirt B, Doll C, Hofmann E, Nahles S, et al. A modified pull-through approach with a pedicled bone flap for oral and oropharyngeal cancer resection: a feasibility study. Surg Radiol Anat. 2024;46:341–52. 38361154 10.1007/s00276-024-03302-3 PMC10960749

[2] Cammaroto G, Stringa LM, Zhang H, Capaccio P, Galletti F, Galletti B, et al. Alternative applications of trans-oral robotic surgery (TORS): a systematic review. J Clin Med. 2020;9: 201. 31940794 10.3390/jcm9010201 PMC7019293

[3] Weinstein GS, O’Malley BW, Rinaldo A, Silver CE, Werner JA, Ferlito A. Understanding contraindications for transoral robotic surgery (TORS) for oropharyngeal cancer. Eur Arch Otorhinolaryngol. 2015;272:1551–2. 25327689 10.1007/s00405-014-3331-9

[4] Cheng SJ, Ko HH, Lee JJ, Kok SH. Comparison of long-term outcomes between pull-through resection and mandibular lip-split surgery for T4a tongue/floor of mouth cancers. Head Neck. 2018;40:144–53. 29140581 10.1002/hed.24994

[5] Li H, Li J, Yang B, Su M, Xing R, Han Z. Mandibular lingual release versus mandibular lip-split approach for expanded resection of middle-late tongue cancer: a case-control study. Journal of Cranio-Maxillofacial Surgery. 2015;43:1054–8. 26116305 10.1016/j.jcms.2015.05.008

[6] Korioth TWP, Romilly DP, Hannam AG. Three-dimensional finite element stress analysis of the dentate human mandible. Am J Phys Anthropol. 1992;88:69–96. 1510115 10.1002/ajpa.1330880107

[7] Pécora JD, Sousa Neto MD, Saquy PC. Internal anatomy, direction and number of roots and size of human mandibular canines. Braz Dent J. 1993;4:53–7. 8180486

[8] Couoh LR, Bucio L, Ruvalcaba JL, Manoel B, Tang T, Gourrier A, et al. Tooth acellular extrinsic fibre cementum incremental lines in humans are formed by parallel branched sharpey’s fibres and not by its mineral phase. J Struct Biol. 2024;216: 108084. 38479547 10.1016/j.jsb.2024.108084

[9] Orassi V, Fischer H, Duda GN, Heiland M, Checa S, Rendenbach C. Silico biomechanical evaluation of WE43 magnesium plates for mandibular fracture fixation. Front Bioeng Biotechnol. 2022;9: 803103. 35223813 10.3389/fbioe.2021.803103 PMC8866862

[10] Ruf P, Orassi V, Fischer H, Steffen C, Duda GN, Heiland M, et al. Towards mechanobiologically optimized mandible reconstruction: CAD/CAM miniplates vs. reconstruction plates for fibula free flap fixation: a finite element study. Front Bioeng Biotechnol. 2022;10:1005022. 36466355 10.3389/fbioe.2022.1005022 PMC9712730

[11] Schwartz-Dabney CL, Dechow PC. Variations in cortical material properties throughout the human dentate mandible. Am J Phys Anthropol. 2003;120:252–77. 12567378 10.1002/ajpa.10121

[12] Lovald ST, Wagner JD, Baack B. Biomechanical optimization of bone plates used in rigid fixation of mandibular fractures. J Oral Maxillofac Surg. 2009;67:973–85. 19375006 10.1016/j.joms.2008.12.032

[13] Lakatos É, Magyar L, Bojtár I. Material Properties of the Mandibular Trabecular Bone. J Med Eng. 2014;2014:470539. 10.1155/2014/470539 PMC4782746 27006933

[14] Schileo E, Taddei F, Cristofolini L, Viceconti M. Subject-specific finite element models implementing a maximum principal strain criterion are able to estimate failure risk and fracture location on human femurs tested in vitro. J Biomech. 2008;41:356–67. 18022179 10.1016/j.jbiomech.2007.09.009

[15] Mirzaali MJ, Schwiedrzik JJ, Thaiwichai S, Best JP, Michler J, Zysset PK, et al. Mechanical properties of cortical bone and their relationships with age, gender, composition and microindentation properties in the elderly. Bone. 2016;93:196–211. 26656135 10.1016/j.bone.2015.11.018

[16] Ugural AC, Fenster SK. Advanced strength and applied elasticity. 4th ed. Upper Saddle River, NJ: Prentice Hall Professional Technical Reference; 2003. p. 152.

[17] Gerhards F, Kuffner HD, Wagner W. Pathological fractures of the mandible. A review of the etiology and treatment. Int J Oral Maxillofac Surg. 1998;27:186–90. 9662010 10.1016/s0901-5027(98)80007-6

[18] van Eijden TM. Biomechanics of the mandible. Crit Rev Oral Biol Med. 2000;11:123–36. 10682903 10.1177/10454411000110010101

[19] Ritchie RO, Kinney JH, Kruzic JJ, Nalla RK. A fracture mechanics and mechanistic approach to the failure of cortical bone. Fatigue Fract Eng Mater Struct. 2005;28:345–71.

[20] Bujtar P, Simonovics J, Váradi K, Sándor GKB, Pan J, Avery CME. Refinements in osteotomy design to improve structural integrity: a finite element analysis study. Br J Oral Maxillofac Surg. 2013;51:479–85. 23084459 10.1016/j.bjoms.2012.09.015

[21] Mattheck C. Design in nature. Interdiscip Sci Rev. 1994;19:298–314.

[22] Sancar B, Çetiner Y, Dayı E. Evaluation of the pattern of fracture formation from trauma to the human mandible with finite element analysis. Part 1: symphysis region. Dent Traumatol. 2023;39:352–60. 36807491 10.1111/edt.12825

[23] Steffen C, Duda K, Wulsten D, Voss JO, Koerdt S, Nahles S, et al. Clinical and technical validation of novel bite force measuring device for functional analysis after mandibular reconstruction. Diagnostics. 2023;13:586. 36832074 10.3390/diagnostics13040586 PMC9955263

[24] Singh G, Mishra M, Gaur A, Pathak D. Comparison of bite force in patients after treatment of mandibular fractures with 3-dimensional locking miniplate and standard miniplates. Traumaxilla. 2019;1:7–10.

