# Supplementary material for: Impact of osteotomy angle on bone failure risk in a modified pull-through approach: a finite element analysis
Source: BMC Oral Health. 2025 Sep 8;25:1403. doi: 10.1186/s12903-025-06732-6 (PMC12418665; doi:10.1186/s12903-025-06732-6)
Supplement: Supplementary file 1 — Supplementary Material [file 12903_2025_6732_MOESM1_ESM.docx]

## SUPPLEMENTARY DATA

# S1. Convergence tests

Four different mesh sizes were tested for each osteotomy scenario. The average edge length for the whole mandible was approximately 1.0-1.5 mm. The ranges of edge lengths for each mesh size in the symphysis are reported in Tables S1-4. The median maximum principal strain values were calculated within the cortical bone of the mandibular symphysis for all cases. Mesh sizes with strain values deviating by less than 2% from the finest mesh were selected for this study.

**Table S1.** Mesh size, median maximum principal strain (ε), and percentage error relative to the finest mesh for wedge osteotomy W1. Mesh C was selected for the study based on the convergence criterion (error < 2%).

| **W1** | **Number of Elements** | **Edge Lengths (mm)** | **Median(ε) (-)** | **Error (%)** |
| --- | --- | --- | --- | --- |
| Mesh A | 91915 | 0.6-0.7 | 1.10E-05 | 9.8 |
| Mesh B | 158272 | 0.5-0.6 | 1.31E-05 | 6.8 |
| **Mesh C** | **313552** | **0.3-0.4** | **1.23E-05** | **0.4** |
| Mesh D | 595471 | 0.2-0.3 | 1.22E-05 | 0.0 |

**Figure S1.** Mesh convergence graph for wedge osteotomy W1.

**Table S2.** Mesh size, median maximum principal strain (ε), and percentage error relative to the finest mesh for wedge osteotomy W2. Mesh C was selected for the study based on the convergence criterion (error < 2%).

| **W2** | **Number of Elements** | **Edge Lengths (mm)** |  | **Median(ε) (-)** | **Error (%)** |
| --- | --- | --- | --- | --- | --- |
| Mesh A | 94981 | 0.6-0.7 |  | 1.47E-05 | 26.9 |
| Mesh B | 219202 | 0.5-0.6 |  | 1.46E-05 | 27.8 |
| **Mesh C** | **480849** | **0.3-0.4** |  | **1.99E-05** | **1.4** |
| Mesh D | 701023 | 0.2-0.3 |  | 2.02E-05 | 0.0 |

**Figure S2.** Mesh convergence graph for wedge osteotomy W2.

**Table S3.** Mesh size, median maximum principal strain (ε), and percentage error relative to the finest mesh for wedge osteotomy W3. Mesh C was selected for the study based on the convergence criterion (error < 2%).

| **W3** | **Number of Elements** | **Edge Lengths (mm)** |  | **Median(ε) (-)** | **Error (%)** |
| --- | --- | --- | --- | --- | --- |
| Mesh A | 92254 | 0.6-0.7 |  | 9.76E-06 | 35.4 |
| Mesh B | 210045 | 0.5-0.6 |  | 1.25E-05 | 17.4 |
| **Mesh C** | **520714** | **0.3-0.4** |  | **1.54E-05** | **1.6** |
| Mesh D | 661727 | 0.2-0.3 |  | 1.51E-05 | 0.0 |

**Figure S3.** Mesh convergence graph for wedge osteotomy W3.

**Table S4**. Mesh size, median maximum principal strain (ε), and percentage error relative to the finest mesh for wedge osteotomy W4. Mesh C was selected for the study based on the convergence criterion (error < 2%).

| **W4** | **Number of Elements** | **Edge Lengths (mm)** |  | **Median(ε) (-)** | **Error (%)** |
| --- | --- | --- | --- | --- | --- |
| Mesh A | 89929 | 0.6-0.7 |  | 1.33E-05 | 43.2 |
| Mesh B | 217667 | 0.5-0.6 |  | 1.86E-05 | 20.8 |
| **Mesh C** | **515031** | **0.3-0.4** |  | **2.36E-05** | **0.5** |
| Mesh D | 750028 | 0.2-0.3 |  | 2.35E-05 | 0.0 |

**Figure S4.** Mesh convergence graph for wedge osteotomy W4.


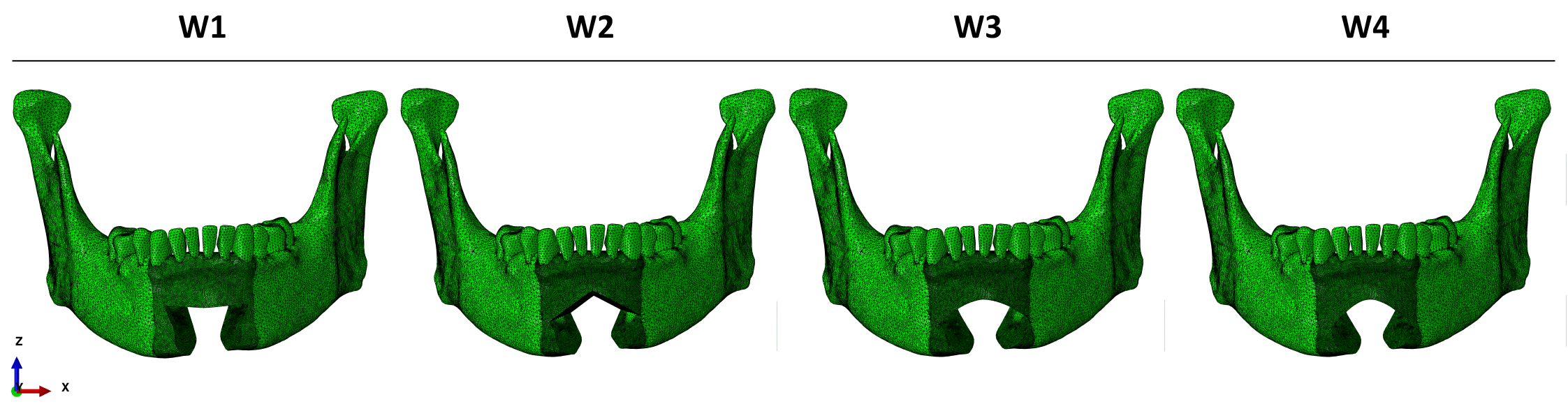


**Figure S5.** Overview of the final mesh sizes after the convergence test in the symphysis region.
